# PEDOT-Coated Red Phosphorus Nanosphere Anodes for Pseudocapacitive Potassium-Ion Storage

**DOI:** 10.3390/nano11071732

**Published:** 2021-06-30

**Authors:** Dan Zhao, Qian Zhao, Zhenyu Wang, Lan Feng, Jinying Zhang, Chunming Niu

**Affiliations:** 1 School of Material Science and Engineering, Shaanxi Key Laboratory of Green Preparation and Functionalization for Inorganic Materials, Shaanxi University of Science and Technology, Xi’an 710021, China; 1902085@sust.edu.cn (Q.Z.); FengLan_0715@163.com (L.F.); 2 Center of Nanomaterials for Renewable Energy, State Key Laboratory of Electrical Insulation and Power Equipment, School of Electrical Engineering, Xi’an Jiaotong University, Xi’an 710054, China; jinying.zhang@mail.xjtu.edu.cn (J.Z.); cniu@xjtu.edu.cn (C.N.); 3 Department of Computational Materials Design, Max-Planck-Insitut für Eisenforschung GmbH, Max-Planck-Strasse 1, D-40237 Düsseldorf, Germany

**Keywords:** red phosphorus, potassium-ion storage, PEDOT, diffusion, pseudocapacitive

## Abstract

Potassium-ion batteries (KIBs) have come up as a potential alternative to lithium-ion batteries due to abundant potassium storage in the crust. Red phosphorus is a promising anode material for KIBs with abundant resources and high theoretical capacity. Nevertheless, large volume expansion, low electronic conductivity, and limited K^+^ charging speed in red phosphorus upon cycling have severely hindered the development of red phosphorus-based anodes. To obtain improved conductivity and structural stability, surface engineering of red phosphorus is required. Poly(3,4-ethylenedioxythiophene) (PEDOT)-coated red phosphorus nanospheres (RPNP@PEDOT) with an average diameter of 60 nm were synthesized via a facile solution-phase approach. PEDOT can relieve the volume change of red phosphorus and promote electron/ion transportation during charge−discharge cycles, which is partially corroborated by our DFT calculations. A specific capacity of 402 mAh g^−1^ at 0.1 A g^−1^ after 40 cycles, and a specific capacity of 302 mAh g^−1^ at 0.5 A g^−1^ after 275 cycles, were achieved by RPNP@PEDOT anode with a high pseudocapacitive contribution of 62%. The surface–interface engineering for the organic–inorganic composite of RPNP@PEDOT provides a novel perspective for broad applications of red phosphorus-based KIBs in fast charging occasions.

## 1. Introduction

Lithium-ion batteries (LIBs) have found wide application in the area of consumer electronics such as portable electronics, electrical vehicles, and grid-scale energy storage [1]. However, owing to the uneven distribution and limited resources of lithium, attention has shifted to other alternative rechargeable battery technologies [2]. Potassium-ion batteries (KIBs) are one of the promising candidates due to the abundance of potassium resources and the low cost of raw materials [3,4,5,6,7]. Considerable efforts have been devoted to searching for applicable anode materials to gain stable and fast K^+^ insertion/extraction [8]. Among the promising anode materials, e.g., carbonaceous materials [9,10,11], titanium-based compounds [12,13], alloys [14,15], and metal oxides/chalcogenides/phosphides [16,17,18], red phosphorus (RP) has attracted increasing attention due to the high abundance of P element and its high theoretical capacity (843 mAhg^−1^ with KP) [19,20].

However, low electronic conductivity and the great volume expansion (>300%) upon cycling have severely limited the large-scale commercial utilization of RP-based anodes [21,22]. Furthermore, the surface-induced pseudocapacitive process, which involves the charge transfer with surface/subsurface atoms [23,24], is imperative to satisfy fast charging demands. It is difficult to achieve high rate performance by the diffusion-controlled pseudocapacitive process due to the large size of K^+^ ions and poor mass diffusion of K^+^ ions in the bulk RP. Reducing the particle size of RP [25,26] and further surface engineering can improve the diffusion kinetics to some extent. Nevertheless, the particle size and surface condition of RP that is synthesized by widely-used vaporization−condensation and mechanical ball-milling techniques are uncontrollable [27,28]. Thus, preparing uniformly distributed RP nanoparticles with a robust surface condition that is suitable for boosting electron/ion diffusion is imperative, which can be achieved by incorporating carbon, alloy, and conjugated polymer [29,30,31,32,33] surface layers.

Herein, RP nanospheres wrapped by Poly(3,4-ethylenedioxythiophene) (PEDOT) as volume expansion buffering and electrical conductivity boosting layer were synthesized through a facile solution-phase approach. PEDOT was coated onto RP nanoparticles to yield RPNP@PEDOT particles with well-controlled morphology and size distribution (average diameter of around 60 nm) [34]. The small size of RP nanospheres can shorten the transport distance of electrons and K^+^ during insertion/extraction. In addition, a continuous and fast electron and ion path in the electrode can be provided by PEDOT [29,35,36,37]. Moreover, PEDOT conformal coating could relieve the volume variation and pulverization of RP nanoparticles upon cycling, and thus enhance the structural stability [36]. As a consequence, the produced RPNP@PEDOT hybrid material exhibits superior rate capability, outstanding pseudocapacitive behavior, and ultra-high capacity than bare RP nanospheres when utilized as KIB anodes. For this reason, a specific capacity of 402 mAh g^−1^ at 0.1 A g^−1^ after 40 cycles, and a specific capacity of 302 mAh g^−1^ at 0.5 A g^−1^ after 275 cycles, were achieved by RPNP@PEDOT anode, which stands out from other reported phosphorus-based KIB anodes, showing strong competitiveness in potassium storage fields. The pseudocapacitive contribution for KIB is 62%, showing great potential in rapid charging. The combined organic–inorganic coating design provides a new insight to improve the electrochemical performance of RP-based nanocomposites, which are promising anode materials for secondary batteries.

## 2. Materials and Methods

### 2.1. Preparation of Materials

**Figure 1:**
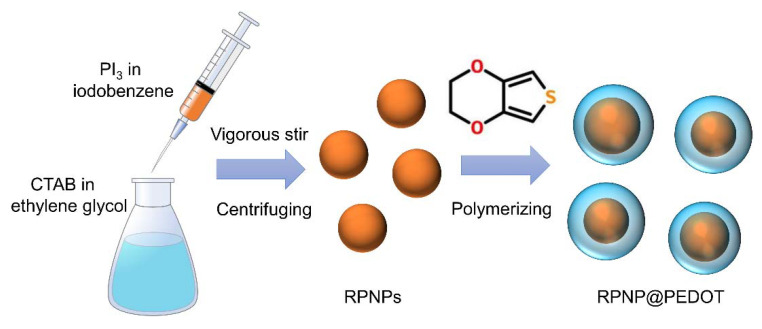
Schematic illustration of synthesis procedure of RPNPs and RPNP@PEDOT.

A solution-phase approach has been utilized to synthesize red phosphorus nanospheres (RPNPs) on a large scale in an ambient environment (shown in Figure 1). A total of 3.09 g of PI_3_ was reduced by 10 mL ethylene glycol to generate RPNPs in the presence of 0.016 mol/L CTAB by vigorous stirring for less than 10 min. CTAB was utilized as a surfactant to limit the growth of phosphorus. After centrifuging at 8000 rpm and drying in a vacuum overnight, RPNPs were obtained and stored in a glove box filled with Ar. To coat PEDOT onto RPNPs, EDOT was added to HCl solution and stirred vigorously for 0.5 h. Then, RPNPs and (NH_4_)_2_S_2_O_8_ were added to the solution and stirred for another 10 h. The resulting orange powder was washed several times with distilled water and ethanol, and dried overnight at 80 °C to get RPNP@PEDOT.

### 2.2. Characterization

Raman spectroscopies were obtained by a single monochromator with a microscope (Reinishaw inVia) equipped with CCD array detector, and 514 nm Argon ion laser was selected for sample excitation. The morphologies of RPNPs and RPNP@PEDOT were identified by field emission scanning electron microscopy (FESEM, SU8100, HITACHI) and high-resolution transmission electron microscopy (HRTEM, FEI Tecnai G2 F20 S-TWIN). X-ray diffraction (XRD) patterns were obtained using a Bruker equipment. The XPS spectra of RPNPs and RPNP@PEDOT were obtained by AXIS SUPRA.

### 2.3. Electrochemical Measurements

The K^+^ storage performance of RPNP@PEDOT and its control sample, RPNPs were measured with a half-cell KIB configuration. The CR2025 coin-type cells were assembled in an argon-filled glove box with both moisture and oxygen levels less than 0.1 ppm (Etelux Minilab glove box). K plates with diameters of 12 mm were used as counter electrodes. The work electrodes were prepared by mixing the active materials (80 wt%), super-P (10 wt%), and poly (vinyl difluoride) (PVDF, 10 wt%) pasting on a pure Cu foil for both KIB cell. Glass fiber (Whatman) films were used as separators for KIBs. The electrolyte used for KIBs was 1.0M KFSI in a 50:50 (*v*/*v*) mixture of ethylene carbonate (EC) and diethyl carbonate (DEC). The galvanostatic charge and discharge experiments were performed on a NEWARE multi-channel battery test system in the voltage range between 0.01 and 2.00 V vs. K/K^+^. The cyclic voltammetry (CV) profiles were obtained from the Autolab PGSTAT302N electrochemical workstation in the voltage range of 0.01–2.00 V vs. K/K^+^ at a scanning rate of 0.1 to 2.0 mV s^−1^.

### 2.4. Calculation Method

All our calculations were performed using the plane-wave Vienna Ab initio Simulation Package (VASP, version 5.4.4) [38,39,40]. A projector augmented-wave (PAW) pseudopotential method [41] was applied to describe interactions between core and valence electrons. A kinetic energy cutoff of 500 eV and Γ-centred 2 × 2 × 2, 2 × 2 × 3 and 2 × 2 × 1 k-point meshes were adopted for PEDOT, RP and PEDOT/RP heterostructure, respectively. These parameters were necessary for convergence of the total energy to within 10^−5^ eV per atom and force less than 0.01 eV/Å per atom. The PBEsol functional [42], a version of Perdew–Burke–Ernzerh (PBE) functional revised for solids was used for geometry optimization. The climbing image nudged elastic band (CI-NEB) method [43,44], an efficient method in determining the minimum energy diffusion path between two given positions, was applied to estimate the energy barrier for diffusion of K^+^.

## 3. Results and Discussion

### 3.1. Morphology and Structure of RPNP@PEDOT

**Figure 2:**
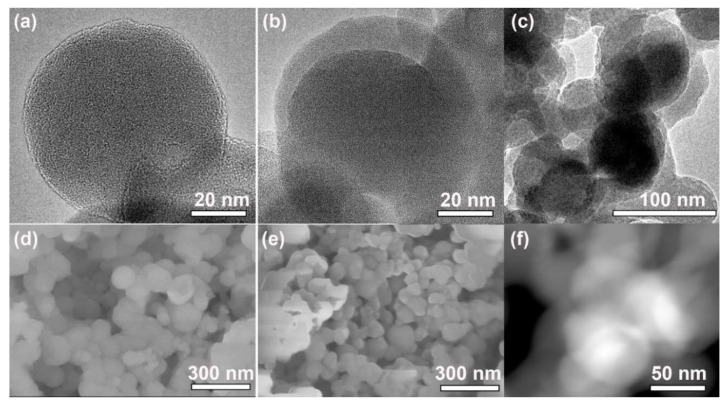
TEM images of (**a**) RPNPs and (**b**) RPNP@PEDOT; SEM images of (**d**) RPNPs and (**e**) RPNP@PEDOT; (**c**) wide-range TEM picture of RPNP@PEDOT and (**f**) the corresponding HAADF image of RPNP@PEDOT.

The RPNPs with an average diameter of 60 nm were prepared by a facile solution-phase oxidation-reduction reaction (Figure 2a,d). After polymerization of EDOT, a conformal PEDOT layer of ~15 nm is uniformly coated onto the RPNPs, forming a hierarchical RPNP@PEDOT core-shell structure (Figure 2b,e). The wide-range TEM picture of RPNP@PEDOT is shown in Figure 2c. The corresponding HAADF image of Figure 2c is shown in Figure 2f, in which the RPNP@PEDOT particles were deformed to some extent due to relatively long-time electron irradiation during the HAADF process. Nevertheless, the contrast between the PEDOT layer and the inner RPNPs is still clear to be distinguished, further confirming the successful wrapping of PEDOT onto RPNPs.

**Figure 3:**
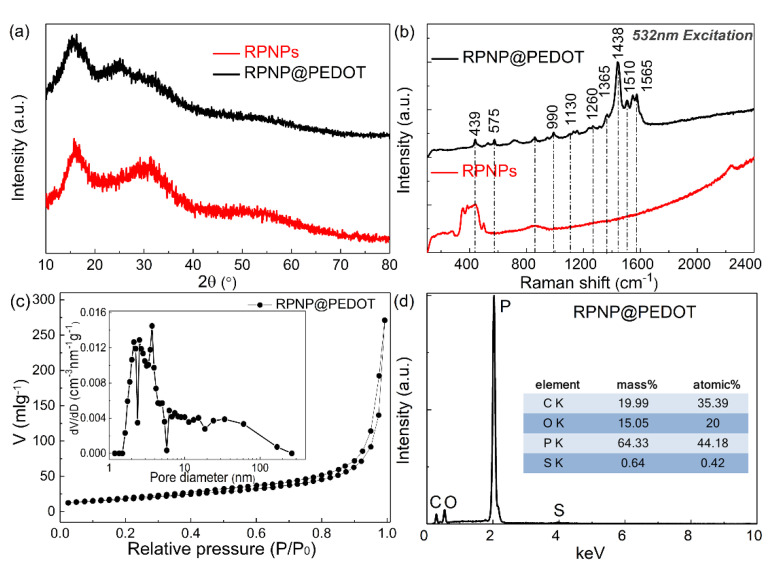
(**a**) XRD patterns and (**b**) Raman spectra of RPNPs (red) and RPNP@PEDOT (black); (**c**) Nitrogen adsorption and desorption isotherms and (**d**) SEM-EDS elemental analysis for RPNP@PEDOT.

The measured XRD pattern of bare RPNPs and RPNP@PEDOT are similar to each other (Figure 3a) and dominated by three broadened diffraction peaks at 13−16°, 25−38°, and 47−65°, respectively, which is consistent with the XRD patterns of typical RP-based materials. The RP-related peak intensity in the XRD pattern of RPNP@PEDOT is weakened because of the PEDOT polymer coating layer. The measured Raman features of RPNPs are consistent with reported commercial RP, which contains a broadband within 300 to 500 cm^−1^. After coating PEDOT, the observed bands in the Raman spectra of RPNP@PEDOT (Figure 3b) are all attributable to PEDOT, further indicating the successful polymerization of the PEDOT layer. In particular, the strong peak located at 1438 cm^−1^ is associated with the symmetric stretching vibration of C=C. Compared with standard PEDOT data, the blue shift of this peak indicates good doping during the synthesis process, which enhances the conductivity of the polymer. The peak located at 1510 cm^−1^ is associated with the asymmetric C=C stretching vibrations.

The N_2_ adsorption–desorption isotherms and calculated BJH pore-size distributions of RPNP@PEDOT are shown in Figure 3c. The specific surface area of RPNP@PEDOT is 64.88 m^2^ g^−1^, which was determined from the linear portion of the Brunauer–Emmett–Teller (BET) plot. The decomposition temperature of RP (within 300–400 °C) is close to that of PEDOT. Thus, TGA measurement is unable to determine the mass content of PEDOT in RPNP@PEDOT. The SEM-EDS analysis was utilized to estimate the phosphorus content in RPNP@PEDOT. To reduce the analysis error of carbon content, RPNP@PEDOT was dispersed onto a silicon wafer other than directly attached on conductive adhesive during SEM-EDS measurements. As shown in Figure 3d, the phosphorus content is ~64.33 wt% in the RPNP@PEDOT sample.

**Figure 4:**
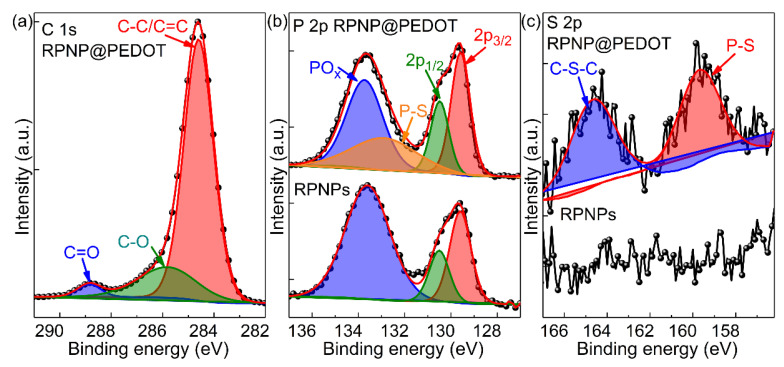
XPS spectrum of RPNPs and RPNP@PEDOT: (**a**) C 1s, (**b**) P 2p, and (**c**) S 2p.

To further characterize the surface chemical composition of RPNPs and RPNP@PEDOT samples, XPS measurements were conducted. As shown in Figure 4a, the C 1s spectra of RPNP@PEDOT show three peaks at 284.6, 285.8, 288.9 eV, which are associated with C–C/C=C, C–O, C=O bonds in the PEDOT surface layer, respectively. In the XPS spectra of P 2p from RPNPs and RPNP@PEDOT (Figure 4b), the doublet located at 129.6 and 130.5 eV (split by 0.85 eV with an integrated intensity ratio of 2:1) corresponds to P 2p_3/2_ and P 2p_1/2_ orbitals, which are consistent with reported XPS data of RP. In addition, the peak located at 133.8 eV is due to the oxidation of RP and the formation of PO_x_. RP, especially nano-sized RPNPs, is prone to oxidation in the ambient atmosphere. Compared with RPNP@PEDOT, the PO_x_ peak is much stronger in the XPS P 2p spectra of RPNPs, indicting the oxidation protection and stabilization functions of the PEDOT layer for RPNPs. In the S 2p spectrum of RPNP@PEDOT (Figure 4c), two weak peaks located at 164.6 and 159.6 eV correspond to C–S–C and P-S bonds, respectively, indicating the formation of a phosphorus–sulfur bond during the liquid-phase reaction. In contrast, no visible S 2p signal was detected in the RPNPs sample.

### 3.2. Potassium Ion Battery Anode Performance

**Figure 5:**
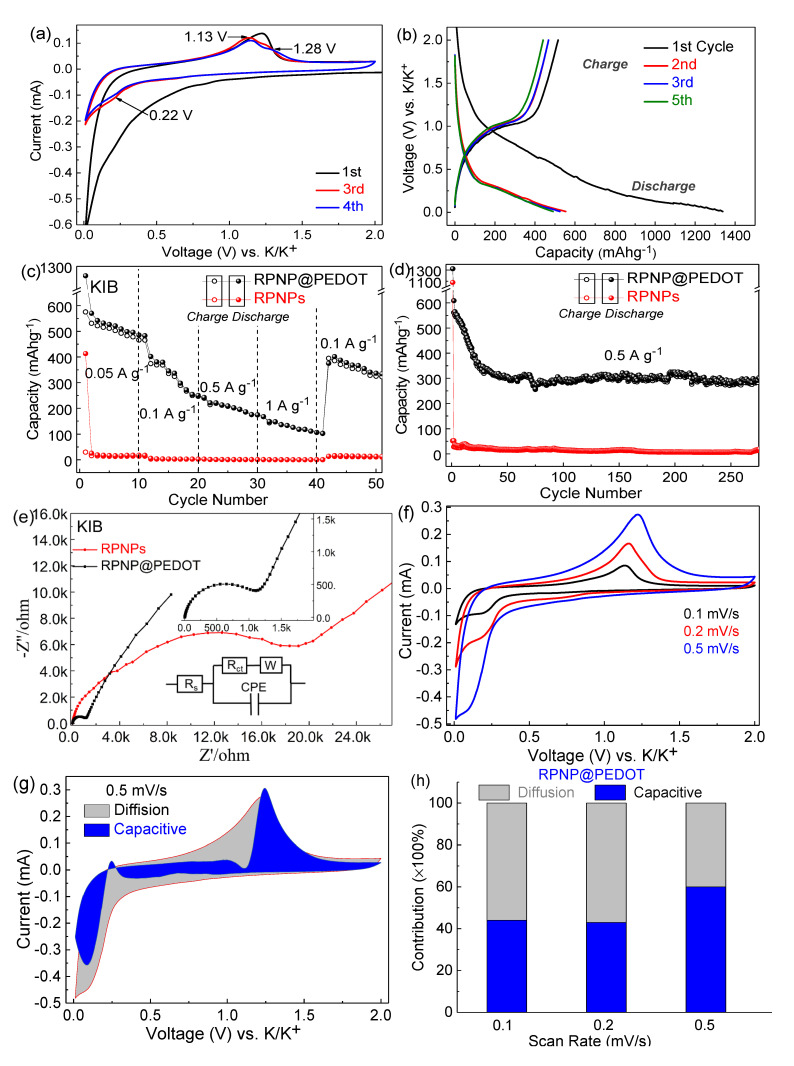
KIB performances of RPNPs and RPNP@PEDOT anodes. (**a**) CV profile at a scan rate of 0.2 mV s^−1^; (**b**) Charge-discharge voltage profiles at 0.1 A g^−1^; (**c**) Rate capacities; (**d**) Cycling performance of RPNP@PEDOT anodes at 0.5 A g^−1^; (**e**) Nyquist plots from 0.01 Hz to 100 kHz; (**f**) CV curves of RPNP@PEDOT anode at different scan rates; (**g**) Capacitive and diffusion current contributions to the charge storage of RPNP@PEDOT at a scan rate of 2.0 mV s^−1^. (**h**) Charge contributions from capacitance and diffusion at various scan rates for RPNP@PEDOT anode.

The electrochemical performances of RPNPs and RPNP@PEDOT as anodes for KIBs were investigated using a half-cell configuration. The CV profile at a scan rate of 0.2 mV s^−1^ and charging–discharging profiles at a current density of 0.1 A g^−1^ of RPNP@PEDOT are shown in Figure 5a,b to elaborate the K^+^ insertion/extraction mechanism. The CV peaks agree well with the correlative potential plateaus of the discharge/charge voltage profiles of RPNP@PEDOT anodes. The peak positions are consistent with most reported RP-based KIB anodes. In the CV curve of the RPNP@PEDOT KIB anode, an unrepeatable major peak at ~0.01 V (vs. K/K^+^) was observed in the first cathodic scan, which is associated with the SEI formation. A weak cathodic peak located at 0.22 V appeared during the subsequent cycles, which represents the K^+^ insertion reaction. Two oxidation peaks centered at 1.13 and 1.28 V appeared in the anodic scans due to the depotassiation processes of RP and PEDOT.

The rate performance of RPNP@PEDOT and RPNPs for KIB half-cells is illustrated in Figure 5c. The KIB performances (capacities and stability) of RPNP@PEDOT anodes are much better than those of RPNPs counterparts due to the structural protection and increased electrical conduction induced by PEDOT. The 1st discharge capacity of RPNP@PEDOT and RPNPs is 1137 and 413 mAh g^−1^ (at 50 mA g^−1^), respectively. The 2nd-cycle discharge capacities of 570, 402, 222, and 150 mAh g^−1^ were obtained from RPNP@PEDOT anodes at current rates of 0.05, 0.1, 0.5, and 1 A g^−^^1^, respectively, showing excellent rate performance. After changing the current back to 0.1 A g^−1^ after 40 cycles, the capacity recovered to 402 mA h g^−1^, exhibit excellent reversible performance. By contrast, the 2nd-cycle discharge capacities of 18, 3, 1, 1, and 13 mAh g^−1^ were obtained from RPNPs anodes at current rates of 0.05, 0.1, 0.5, 1, and 0.1 A g^−1^, respectively.

The KIB cycling stability at 0.5 A g^−1^ is shown in Figure 5d. The 1st discharge capacity of RPNP@PEDOT and RPNPs are 1322 and 1078 mAh g^−1^, respectively, confirming the potassium storage capability of RP. Capacity degradation was observed in the first 30 cycles in the case of RPNP@PEDOT, which is a common phenomenon for phosphorus-based anodes. After the limited degradation process, the capacity of RPNP@PEDOT stabilized at 302 mAh g^−1^ after 275 cycles at 0.5 A g^−1^, showing excellent long-term durability and fast-charging potential. As a contrast, huge capacity degradation (from 1078 to 53 mAh g^−1^) occurred at the 2nd-cycle in the case of RPNPs, and the capacity stabilized at only ~20 mAh g^−1^ at the final stage. The poor potassium storage ability of bare RPNPs is probably due to the undesirable surface condition of bare RPNPs for K^+^ to transport.

The electrochemical impedance spectroscopy (EIS) spectra were measured and shown by Nyquist plots (Figure 5e). In the inset equivalent circuit model, R_s_, R_ct_, CPE, and W are the resistance between current collector and electrolyte, charge transfer resistance, constant phase element for double-layer capacitor, and Warburg impedance, respectively. The fitted R_ct_ of RPNP@PEDOT anode (1110 Ω) is much lower than that of RPNPs anode (~20,000 Ω). Additionally, the slope of −Z’’ vs. Z’ curve at the low-frequency range for the RPNP@PEDOT anode is higher than that of the RPNPs anode, suggesting more efficient K^+^ diffusion and corresponding pseudocapacitance behavior of the RPNP@PEDOT.

To further investigate the pseudocapacitance behavior of the RPNP@PEDOT hybrid, the charge storage kinetics of the RPNP@PEDOT and RPNPs were evaluated by CV measurements at various scan rates (Figure 5f). The capacitive-controlled contribution ratio and diffusion-controlled contribution ratio in the RPNP@PEDOT and RPNPs anodes were quantitatively acquired using the measured CV curves and Equation (1):

(1) $$i=k_{1}v+k_{2}v^{\frac{1}{2}}$$

where i and v are current density and scan rate, respectively. The k_1_ and k_2_ are constants related to capacitive contribution and diffusion contribution of the current response in the CV curves. Thus, the current at a fixed potential (V) is composed of two kinetic processes: capacitive ($k_{1}v$) and diffusion-controlled ($k_{2}v^{\frac{1}{2}}$) processes. The capacitive contribution of the RPNP@PEDOT at a scan rate of 0.5 mV s^−1^ is 62%, as illustrated in Figure 5g (blue color), and the remaining grey color region is diffusion-dominated. The capacitive contributions of the RPNP@PEDOT anode are 62%, 43%, and 44% at 0.5, 0.2, and 0.1 mV s^−1^, respectively (shown in Figure 5h), showing considerable pseudocapacitive K^+^ storage behavior.

**Figure 6:**
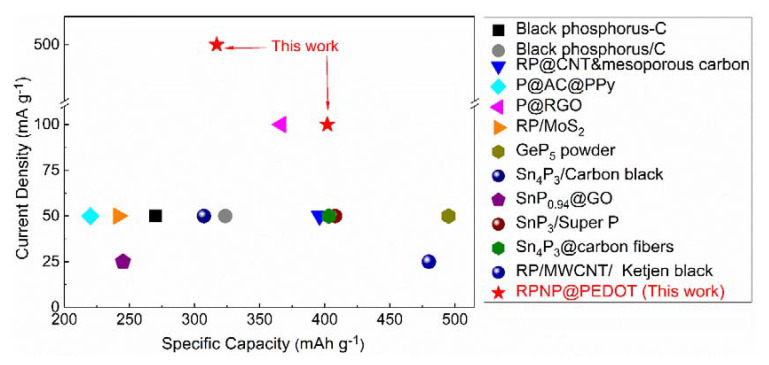
KIB performance comparisons of various phosphorus-based anodes. The specific capacity figures are reliable data after cycling at least 40 cycles.

The potassium storage performance of the RPNP@PEDOT was compared with various phosphorous/carbon-based hybrids in terms of depotassiation capacity, as shown in Figure 6 and Table 1. The outstanding cycling performance of the RPNP@PEDOT can be ascribed to the strong structural stabilization effect of the PEDOT coating layer during insertion/extraction of K^+^ ions, whereas, the distinguished rate capacity and pseudocapacitive behavior benefit from the enhanced electron and ion conductivity induced by PEDOT.

**Figure 7:**
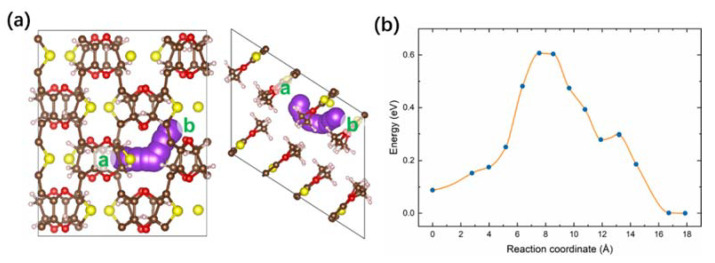
(**a**) Schematic diagram of diffusion pathway in the PEDOT, and (**b**) the energy profile of the K diffusion from sites a to b.

**Figure 8:**
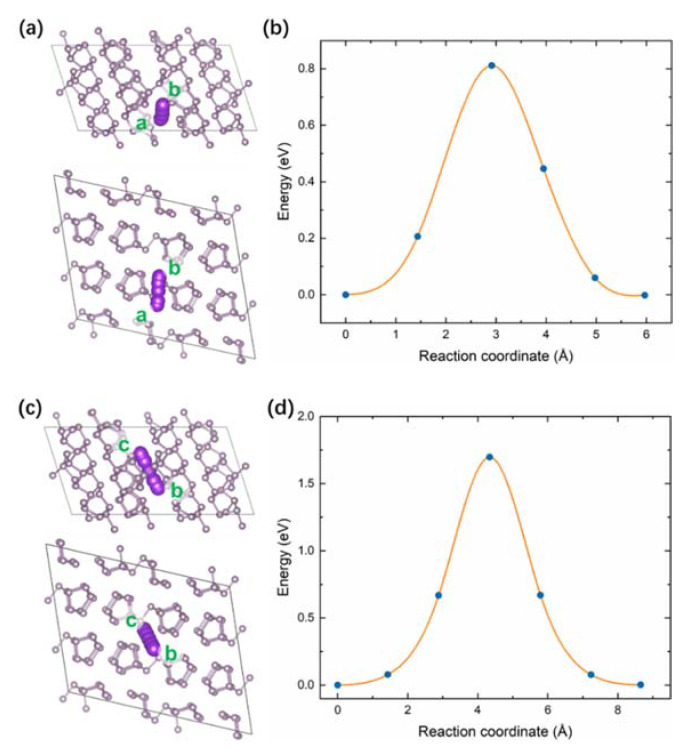
Schematic diagram of diffusion pathway in the RP (**a**) from site a to b, and (**c**) from site c to b. The energy profile of the K^+^ diffusion of the two pathways is shown in (**b**,**d**), respectively.

To further elucidate the improved electrochemical properties of the RPNP@PEDOT hybrid, DFT calculations were performed to investigate the diffusion behavior of K^+^ in the PEDOT and RP. The climbing image nudged elastic band (CI-NEB) method was applied to study the diffusion of a single K ion in the PEDOT and RP by calculating the variation in energy as K^+^ moves between equivalent interstitial sites. The diffusion coordinate is calculated based on the cumulative sum of the trajectory length of all atoms in the structures. Figure 7a shows the trajectory of K^+^ diffusion in the PEDOT molecule, while the corresponding energy profiles along pathways (a to b sites) are exhibited in Figure 7b. This pathway involves an energy barrier of 0.61 eV. In terms of K^+^ diffusion in the RP, two pathways were considered. One is parallel to the phosphorus layers (Figure 8a), whereas the other one is across the phosphorus layers (Figure 8c). Pathways from a to b sites have a lower energy barrier of 0.81 eV (Figure 8b). However, as K^+^ moves from c to d sites, it passes across a P–P bond by overcoming a higher energy barrier of 1.70 eV (Figure 8d). Compared with the diffusion barriers in the RP, the one in the PEDOT module is lower, indicating higher K^+^ mobility, which will help to avoid the accumulation of K ions on the surface and facilitate the storage of K ions when taking it as an anode.

## 4. Conclusions

The RPNP@PEDOT hybrid with a core-shell structure was synthesized by a simple liquid-phase procedure, and characterized by HRTEM, SEM, XRD, XPS and Raman scattering. The PEDOT coating can prevent the RPNPs from pulverization and fragmentation into the electrolyte during potassiation/depotassiation cycles. In addition, P–S bonding at the interface of the PEDOT and RPNPs was verified by XPS characterization, which can further stabilize the core-shell structure upon cycling. Enhanced electron and ion transportation were induced by the PEDOT due to its conjugated polymer nature. This is partially evidenced by our DFT calculation in terms of the lower diffusion barriers of K^+^ in the PEDOT than that in the RP. The improved electrochemical performances of the RPNP@PEDOT anode than the bare RPNPs anode are attributed to the unique inorganic-organic combination structural design. As a result, a specific capacity of 402 mAh g^−1^ at 0.1 A g^−1^ after 40 cycles, and a specific capacity of 302 mAh g^−1^ at 0.5 A g^−1^ after 275 cycles, was achieved by the RPNP@PEDOT anode, indicating its excellent potassium storage properties.

## Figures and Tables

**Table 1:** Comparison of the potassium storage performance of various phosphorous/carbon-based hybrids.

Material	Synthetic Method	Cycling Stability (after n Cycles)	References
Black phosphorus-C	ball-milling	270 mAh g^−1^ at 50 mA g^−1^ after 50 cycles	[5]
Black phosphorus/C	ball-milling	323.5 mA h g^−1^ at 50 mA g^−1^ after 50 cycles	[45]
RP/MWCNT/Ketjen black	Wet-ball milling	480 mA h g^−1^ at 25 mA g^−1^ after 50 cycles (recalculated based on the whole electrode mass)	[20]
RP@CNT mesoporous carbon	vaporization/condensation	396 mAh g^−1^ at 50 mA g^−1^ after 75 cycles	[46]
P@AC@PPy	vaporization/condensation	220 mAh g^−1^ at 50 mA g^−1^ after 200 cycles	[8]
P@RGO	vaporization/condensation	366.6 mAh g^−1^ at 100 mA g^−1^ after 50 cycles	[47]
RP/MoS_2_	ball-milling	241.4 mAhg^−1^ at 50 mAg^−1^ after 120 cycles	[48]
GeP_5_ powder	ball-milling	495.1 mAh g^−1^ at 50 mA g^−1^ after 50 cycles	[49]
Sn_4_P_3_/Carbon black	ball-milling	307.2 mAh g^−1^ at 50 mA g^−1^ after 50 cycles	[50]
SnP_0.94_@GO	hot-injection	245 mA h g^−1^ at 25 mA g^−1^ after 50 cycles.	[51]
SnP_3_/Super P	ball-milling	408 mAh g^−1^ at 50 mA g^−1^ after 50 cycles	[52]
Sn_4_P_3_@carbon fibers	Ball-milling; electrospinning	403.1 mAh g^−1^ at 50 mA g^−1^ after 200 cycles	[53]
RPNP@PEDOT	solution phase	402 mAh g^−1^ at 100 mA g^−1^ after 50 cycles of rate testing at various current rates; 302 mAh g^−1^ at 500 mA g^−1^ after 275 cycles of rate testing at various current rates	This work
